# Sheldrake on the "Personal Appearance" of Young Ladies

**Published:** 1829-04-01

**Authors:** 


XLVI.

SHELDRAKE ON THE "PERSONAL APPEARANCE" OF YOUNG LADIES.

The poet has assured us, that "partial ill is universal good," and distortions of the spine is a case in point. What though young ladies are as crooked as cork-screws, what though the best part of their existence is spent on a mattrass, or a board, or within the unyielding *ceinture* of a modern instrument-maker, is there not a Sheldrake to counterbalance, and more than counterbalance all these ills? What are the deformities, the halts, and the lamenesses of some thousands of the female sex to the possession of a jewel like Mr. S. who can teach us to dance, and to fiddle, and to skait;—who instructs us in the mode of "keeping our feet under our legs," (a very *seasonable* piece of information in these frosty months); at whose magic *spell*, the hip-joint gambols and frisks with even more vivacity than the shoulder; and the pointed T slides into an equal square!* Both anxious Misses and their still more anxious Mammas may rest in peace now that this Daniel is come to judgment. The "Lecture," which we had the honour of reviewing before, was on dancing; the present is on walking, and being the more general amusement of the two, we cannot pass it altogether sub-silentio.

We were wont in our ignorance to believe, that young folks learned to walk, as ducklings to waddle and puppies to run, from a species of instruction called instinct. We are undeceived by Mr. Sheldrake, who ingeniously observes that "all the different modes of walking, or *moving about*, have been *produced by those habits* which have been *fixed* by the *pursuits in which the parties who use them have been engaged.*" Thus, a weazen-faced clerk will learn to walk from sitting at his desk from ten in the morning till five in the afternoon—an exquisite, from disporting in his cabriolet up Regent or Bond Street—a tradesman's wife, "pursy and big," from sitting behind her good man's counter serving his customers or guarding the till! The novelty of Mr. Sheldrake's notion is undoubted, and the only objection that we know of is the trick which graceless urchins have got into of walking *before* they follow any "pursuits" whatever. In this town children commonly walk at two or three years of age, but seldom engage in business *quite* so young.

Mr. Sheldrake's expedient for giving "at the earliest period they can be communicated" strength to the ligaments and activity to the muscles, is simply this— "The *whole process* that will be necessary, during what may be called this first stage of instruction, will be, first, *to obtain the full power of directing the feet alternately*, and then to practise walking carefully and steadily *upon the line*, for as much time as can be conveniently devoted to it every day; in doing this, it should be strictly required *that the child should keep her eyes* upon her *feet*, to see that she places her foot exactly against the line at every step; *this will give a firmness to her movements which will afterwards be of the greatest consequence.*"

We have heard of coachmen at a rout, or a levee "keeping the line," but of young ladies and gentlemen doing so, never till now. We should think the proceeding admirably calculated to teach them the graceful accomplishment of turning their toes *in*, and strongly recommend it to mammas accordingly. This, with Mr. Sheldrake is a perfect pa-

---

* See Sheldrake's Lecture, in the present Quarterly Number.

nacea against all distortions, both before the seventh year of age, because *until* that time the practice ought to be continued, and afterwards, "because the *course of exercises* (risum teneatis ?) in which they *have been* engaged will have given them habits which will effectually preserve them from such defects." ! We should like to see the in-toed, bandy-legged little wretches, which *such* a five or six years' "course of exercises" would produce ! Falstaff's regiment, "such scarecrows as no eye hath seen," would be nothing to them !

Mr. Sheldrake is not partial to "busks, braces, monitor spinal stays," &c. in which he only re-echoes the sentiments of the profession, but, as usual with minds of his stamp, he cannot be contented with the dictates of common sense, but must rush into utter absurdity. "I likewise advise," says he, "*that all articles of whatever nature, that are added to the female dress*, should be avoided ; I *do not mean the dresses themselves*, for *all* female dresses *I now believe to be harmless, or at least, that they do no lasting injury* to the persons of those that wear them." ! ! ! Kind, considerate man, to allow the ladies some sort of covering, to spare their modesty and warm their skins ! He "does not mean" that "the dresses themselves" should be abandoned, because on mature reflection he believes them to be "harmless." What, nothing more than *harmless*—not even *useful* in preserving the fair against the inclemencies of January, February, and March ? We used to argue, that the ladies dressed too lightly instead of too much ; that they bared their alabaster necks when they ought to have covered them ; that they flitted in muslins, when they ought to have arrayed themselves in cloths. However, we stand corrected. We would earnestly recommend our fair countrywomen to attend to Mr. Sheldrake's warning, and "avoid all articles of *whatever nature*, that are added to their dress." ! A bracelet or a necklace is fraught with peril—a flounce must be perdition. Under the old régime matters were even worse than now, for in those times, fashion had not such rapid changes as at present, and an article of dress when once introduced, we are told by the lecturer, was worn by all,—"*grandmothers, mothers, and daughters, all wore the same dress.*" ! That same dress could not, however, have been *very* inconveniently tight, which allowed the grandmother, mother, and daughter "all" to get into it ! What a very little family party this "tria juncta in uno," must have made !

"The *padding* that is *put in the hollow* of such a person's back" ("some unfortunate customer") "to make the two sides equal, depresses that hollow more and more ; additional padding is put on, the hollow still increases, *till in the end, the sufferer sinks under her complicated misfortune.*"

From the foregoing melancholy portrait, any one would inevitably be led to infer, that the padding which overwhelms "the sufferer," was pig-iron at least, instead of a little soft *wool.* Fancy an unfortunate young creature tottering under the *load* of a little piece of wadding at one side of her dress, and sinking at last, beneath the Titanic *weight* that oppressed her ! The picture is perfectly harrowing to a man "of warm feelings."

The gist of Mr. Sheldrake's treatment in preventing distortions, besides "the manner of using their legs" to which we have already referred, consists in some motions of the arms to which, passing over the tiresome and inflated verbiage of the lecturer, we shall now advert. The "scholar or patient, which ever it may be called," standing upright, is to look at some object before her, extend both her arms in a straight line, and then turn her body half round, "upon the pelvis," till one hand points to the object, and the other directly backwards. Having been in this position for a certain length of time, she is gradually to turn the body half round the reverse way, till the hand that before pointed backwards points forwards, and the other vice versâ. Another "desirable exercise," is to sit on a chair, with her head against the back, extend her arms horizontally "in a line with each other, and then without moving her seat, her feet, or her head and shoulders from the back of the chair, "bend her body sideways, till she carries one of her hands as near to the ground as possible, the other arm being then proportionably raised in the air." A succession of these manœuvres with each arm must be practised, when the charm will be "firm and good."

And these manipulations are to cure or prevent distortions! Mr. Sheldrake's "scholars or patients, whichever they may be called," might do for sign posts with their arms stuck out "in a line with each other," but assuredly they would do for nothing else, unless, like their doctor, or master, whichever he may be called, for laughing-stocks! These lectures are all alike absurd—"fool the second reigns like fool the first," and we leave them with a mixture of contempt and disgust.

---

XLVII.

